# Lung injury promoted by strong inspiratory efforts and breath stacking: impact of ventilation mode

**DOI:** 10.1186/s40635-025-00821-0

**Published:** 2025-10-29

**Authors:** Yasuhiro Norisue, Sunao Usami, Yukie Ito, Muneyuki Takeuchi, Atsushi Kawamura, Ryuichi Nakayama, Naofumi Bunya, Jun Kataoka, Yusuke Endo, Takaharu Itami, Taku Hirokawa, Chihiro Sugita, Hirotaka Takeshima, Airi Takemoto, Miyako Kyogoku, Junki Koike, Shigeki Fujitani, Francesco Mojoli, Taku Miyasho

**Affiliations:** 1 Department of Pulmonary and Critical Care Medicine, Tokyo Bay Urayasu Ichikawa Medical Center, Urayasu, Chiba Japan; 2 Department of Clinical Engineer, Tokyo Bay Urayasu Ichikawa Medical Center, Urayasu, Chiba Japan; 3 Department of Intensive Care Medicine, Osaka Women’s and Children’s Hospital, Osaka, Japan; 4 Department of Critical Care Medicine, National Cerebral and Cardiovascular Center, Osaka, Japan; 5 Department of Intensive Care Medicine, Division of Anesthesiology and Intensive Care Medicine, Jichi Medical University, Tochigi, Japan; 6 Department of Emergency Medicine, Sapporo Medical University School of Medicine, Sapporo, Japan; 7 Department of Critical Care Medicine, Nerima Hikarigaoka Hospital, Tokyo, Japan; 8 Department of Veterinary Medicine, School of Veterinary Medicine, Rakuno Gakuen University, Ebetsu, Japan; 9 Take Atman Inc., Tokyo, Japan; 10 Department of Diagnostic Pathology, St. Marianna University School of Medicine, Kawasaki, Kanagawa Japan; 11 Department of Emergency and Critical Care Medicine, St. Marianna University School of Medicine, Kawasaki, Kanagawa Japan; 12 Department of Anesthesia and Intensive Care, Fondazione IRCCS Policlinico San Matteo, Pavia, Italy

**Keywords:** Breath stacking, Spontaneous effort, Ventilator-induced lung injury, Volume-controlled ventilation, Pressure-controlled ventilation, Patient–ventilator interaction, Atelectrauma, P-SILI, Porcine model

## Abstract

**Background:**

Breath stacking, particularly double triggering, is a common patient–ventilator asynchrony during strong inspiratory effort. It can cause excessive tidal volumes and high transpulmonary pressures, contributing to ventilator-induced lung injury (VILI). The mode-specific consequences of breath stacking induced by strong inspiratory effort remain unclear.

**Methods:**

In a porcine model of minimal lung injury, 17 animals were randomized to volume-controlled ventilation (VCV, *n* = 9) or pressure-controlled ventilation (PCV, *n* = 8). High respiratory drive was induced with continuous CO₂ inhalation, and ventilator settings were dynamically adjusted to maintain a breath stacking ratio of 40–70% of spontaneous efforts. Measurements included airway and transpulmonary pressures, driving pressures, tidal volume, esophageal pressure swings (ΔPes), stress index (SI), respiratory compliance, and histological lung injury. Risk factors for baro/volutrauma were defined by elevated plateau or driving pressures, transpulmonary pressures, or tidal volume >10 mL/kg. Atelectrauma risk was defined by SI < 0.9, negative end-expiratory transpulmonary pressure (PLexp), or vigorous effort (ΔPes > 5 cmH₂O or Pmus > 8 cmH₂O).

**Results:**

VCV animals exhibited higher respiratory rates (44.0 vs. 30.5 breaths/min, *p* = 0.027), whereas PCV resulted in stronger inspiratory efforts (ΔPes 6.1 vs. 4.2 cmH₂O, *p* = 0.015). During breath stacking, VCV produced larger tidal volumes and higher plateau pressures, accumulating more baro/volutrauma risk factors (median 4.0 vs. 0.0, *p* < 0.001). In contrast, PCV animals developed more atelectrauma risk factors (3.0 vs. 1.0, *p* = 0.004). Histological injury scores were comparable, with a non-significant trend toward greater severity in PCV.

**Conclusions:**

Breath stacking under strong inspiratory drive can promote lung injury through distinct mechanisms depending on ventilation mode. VCV was associated with the risk of overdistension, whereas PCV involved vigorous inspiratory effort and potential atelectrauma. Double triggering should be recognized as a clinical warning sign, prompting careful assessment of respiratory drive, inspiratory effort, and ventilator settings.

**Supplementary Information:**

The online version contains supplementary material available at 10.1186/s40635-025-00821-0.

## Introduction

Lung-protective ventilation is central to the management of acute respiratory distress syndrome (ARDS), with strategies such as limiting tidal volume and controlling airway and transpulmonary pressures being essential to reduce ventilator-induced lung injury (VILI) [1]. However, even when conventional lung-protective settings are maintained, injurious patient–ventilator asynchrony can occur, particularly in the presence of strong respiratory drive [2].

One such form of asynchrony is breath stacking, defined as the delivery of two or more consecutive ventilator insufflations with no or minimal intervening exhalation. This includes double triggering, in which a single patient effort results in two consecutive mechanical breaths, and reverse triggering, a form of patient–ventilator interaction in which a ventilator-delivered breath triggers a reflex diaphragmatic contraction that may subsequently provoke an additional breath—leading to breath stacking [3, 4].

In patients with acute respiratory failure, efforts to limit tidal volume are often undermined by strong inspiratory efforts. During volume-controlled ventilation (VCV), spontaneous effort can lead to double triggering and resultant tidal volumes that nearly double the set value. During pressure-controlled ventilation (PCV), the volume of the second inflation depends largely on the strength and duration of the patient’s inspiratory effort, and double triggering does not necessarily deliver a full additional volume. Nonetheless, the interaction between patient-generated negative pressure and ventilator-delivered positive pressure can still produce excessive tidal volumes as well as high transpulmonary pressures. Regardless of the ventilation mode, intense spontaneous efforts can generate regional increases in lung stress, particularly in dependent areas, promoting intra-tidal recruitment and atelectrauma—injury mechanisms distinct from those associated with global lung overdistention [5]. These efforts can also cause occult pendelluft, the redistribution of gas within the lung due to asynchronous regional ventilation [6]. Although deep sedation may mitigate such vigorous effort, it may paradoxically predispose patients to reverse triggering and subsequent breath stacking [7].

Despite wide recognition in clinical practice that strong inspiratory effort often results in breath stacking, how different ventilatory modes influence the development and consequences of this phenomenon remains poorly understood. To address this, we developed a reproducible porcine model using CO₂-induced high respiratory drive to systematically induce frequent double triggering with breath stacking during both VCV and PCV.

We hypothesized that the ventilator mode would modulate both the mechanical and the pathophysiological consequences of breath stacking under high respiratory drive. Using this preclinical model of minimal lung injury, we aimed to compare the characteristics and impact of frequent breath stacking driven by strong inspiratory efforts on lung stress, strain, risk of atelectrauma, and histological injury between volume-controlled and pressure-controlled ventilation.

## Methods

### Animals and lung injury model

Seventeen healthy juvenile domestic pigs (approximately 30 kg [29.2–30.8 kg]; 9 assigned to the VCV group and 8 to the PCV group) were anesthetized with propofol and butorphanol, endotracheally intubated, instrumented, and subjected to a minimal lung injury protocol. This weight range was selected because juvenile pigs of this size are commonly used in preclinical respiratory studies due to their anatomical similarity to human lungs and practical compatibility with ICU equipment [8]. Lung injury was induced using a double-hit model, consisting of saline lung lavage with 1 L of warmed physiological saline, followed by one hour of injurious ventilation with zero end-expiratory pressure (ZEEP) and an inspiratory pressure of 40 cmH₂O [9]. The duration of injurious ventilation was deliberately limited to prevent excessive lung damage that could mask potential differences in ventilator-induced lung injury between modes. The sample size was based on power calculations indicating that 8 animals per group were required to detect expected between-group differences in key physiological variables, including driving pressure and esophageal pressure swings, with sufficient statistical power. One additional animal was included to preserve statistical power in case of data loss or unforeseen events such as premature death during the protocol.

### Ventilation protocol

Following lung injury induction, animals were randomly assigned to receive either VCV or PCV. In the VCV group, the tidal volume was set at 7 mL/kg. In the PCV group, the inspiratory pressure was adjusted to achieve a delivered tidal volume between 6 and 8 mL/kg during the protocolized ventilation. A mandatory respiratory rate of 12 breaths per minute was applied in both groups throughout the study.

In this study, breath stacking was defined and detected based on the BREATHE Criteria, an objective definition developed by Beitler et al. [10]. Double triggering, a specific form of breath stacking, refers to two mechanical breaths delivered in response to a single inspiratory effort. To increase respiratory drive and promote double triggering, continuous inhalation of carbon dioxide (CO_2_) was administered. Both ventilator settings (flow rate, inspiratory time, trigger sensitivity, and PEEP) and the CO₂ flow rate were dynamically adjusted according to a pre-specified protocol (see Supplementary Fig. 1) to achieve a target breath stacking ratio of 40% to 70% of all spontaneous inspiratory efforts. Notably, PEEP was titrated not for oxygenation purposes, but to modulate respiratory effort and achieve the target breath stacking frequency, based on preliminary experiments aimed at optimization of the model. The fraction of inspired oxygen (FiO₂) was titrated to maintain peripheral oxygen saturation (SpO₂) above 96%. Animals were ventilated in the supine position for 3 h. Mechanical ventilation was provided using either the PB840 ventilator (Medtronic, USA) or the SERVO-U ventilator (Getinge, Sweden).

### Measurements and categorization

Physiological data were continuously recorded using a LabChart data acquisition system (ADInstruments, Australia). Recorded variables included airway pressure, volume, esophageal pressure, and flow waveforms. These were analyzed offline to assess breath timing, spontaneous inspiratory efforts, asynchrony patterns, and respiratory system mechanics.

Flow, tidal volume, airway pressure, and esophageal pressure were analyzed at baseline (10 min after the start of protocolized ventilation) and at 1, 2, and 3 h. At each time point, the first three spontaneous breaths without artifacts were selected, and the average values were calculated. For ΔPes, the first three artifact-free breaths exhibiting breath stacking were selected at each time point. ΔPes was defined as the difference between the esophageal pressure baseline and the nadir during the inspiratory phase of each breath and was used to evaluate the magnitude of inspiratory effort. Inspiratory muscle pressure (Pmus) was estimated based on ΔPes using a calculation incorporating chest wall compliance.

Breath stacking was identified by visual inspection of the waveforms, and the proportion of breaths exhibiting breath stacking was calculated over the entire ventilation period. Accordingly, we aimed to capture the tidal volume corresponding to the moment when the lungs were maximally inflated during breath stacking. Any minor exhalation occurring between the two stacked breaths was disregarded, and tidal volume was calculated from the expiratory flow following the second inflation using LabChart. Respiratory rate was determined based on the esophageal pressure waveform, with each negative deflection representing an inspiratory effort.

Inspiratory hold maneuvers were used to assess airway plateau pressure (Pplat), driving pressure (ΔPaw), transpulmonary plateau pressure (PLplat), and transpulmonary driving pressure (ΔPL) in mechanical breaths with and without breath stacking. Expiratory hold maneuvers were performed to determine end-expiratory transpulmonary pressure (PLexp). The following thresholds (average values measured during breath stacking over the 3-h ventilation period) were considered indicators of barotrauma or volutrauma risk: Pplat > 30 cmH₂O, ΔPaw > 15 cmH₂O, PLplat > 20 cmH₂O, ΔPL > 10 cmH₂O, and tidal volume > 10 mL/kg.

Additionally, the stress index (SI) was determined immediately after the 3-h ventilation period, during which all animals were paralyzed and switched to VCV with a constant inspiratory flow. SI was calculated by fitting the airway pressure–time curve during the inspiratory phase to the equation P(*t*) = *a*‧*t*^*b*^ + *c*, where the exponent *b* represents the SI value [8]. For standardization, only the portion of the pressure–time curve corresponding to the first 180 mL of inspired tidal volume was used for analysis.

An SI < 0.9 during passive inflation, and negative end-expiratory transpulmonary pressure (PLexp < −0.5 cmH₂O), were interpreted as indicators of insufficient PEEP. In the context of lung injury, strong inspiratory efforts can promote regional increases in lung stress, intra-tidal recruitment, and atelectrauma, particularly when PEEP is inadequate to prevent expiratory lung collapse. Accordingly, the following thresholds (average values during the 3-h ventilation period) were considered indicators of atelectrauma risk: SI < 0.9, PLexp < −0.5 cmH₂O, ΔPes > 5 cmH₂O, and Pmus > 8 cmH₂O.

### Lung injury assessment

Lung injury associated with high spontaneous inspiratory efforts and breath stacking was assessed based on respiratory mechanics (compliance), gas exchange (PaO₂/FiO₂ ratio), and histological findings. Compliance and PaO₂/FiO₂ values were measured at baseline and again at the end of the 3-h ventilation period, and changes between these two time points were assessed. At the conclusion of the experiment, animals were euthanized by intravenous injection of a lethal dose of pentobarbital, with or without potassium chloride. Lung tissue samples were obtained from the right posterior lobe. Histological evaluation was performed by an experienced board-certified pathologist (J.K.) using a standardized, blinded scoring system. The histological lung injury score included assessments of alveolar hemorrhage, alveolar wall thickening, neutrophilic infiltration, and hyaline membrane formation [11].

### Statistical analysis

Statistical analyses were performed using MedCalc Statistical Software version 22.016 (MedCalc Software Ltd., Ostend, Belgium) and Python version 3.12.1 with the SciPy (version 1.13.0) and Pingouin (version 0.5.4) libraries (Python Software Foundation, USA). Group comparisons were performed using non-parametric tests (Mann–Whitney *U*), and categorical variables were compared using Fisher’s exact test. A two-sided *p*-value < 0.05 was considered statistically significant.

### Ethical approval

All experimental procedures were approved by the Institutional Research Board of Rakuno Gakuen University (approval number: VH17B6) and were conducted in accordance with established ethical guidelines for animal research.

## Results

All 17 animals randomized to either the VCV group (*n* = 9) or the PCV group (*n* = 8) successfully completed the study protocol.

At baseline (i.e., 10 min after lung lavage followed by one hour of injurious ventilation), gas exchange and compliance parameters indicated minimal lung injury. The median PaO₂/FiO₂ ratio at 0 h was 490 [413–510]  mmHg in the VCV group and 364 [276–419] mmHg in the PCV group. Corresponding respiratory system compliance was 16.1 [15.0–20.8] and 19.7 [16.6–22.1] mL/cmH₂O, respectively. There were no statistically significant differences between groups for either variable.

During the 3-h study period, the average set inspiratory pressure above PEEP was 9.6 cmH₂O [7.6–14.0] in the PCV group. PEEP was set at 8.5 cmH₂O [7.9–9.1] and 7.4 cmH₂O [5.9–8.5] in the VCV and PCV groups, respectively (*p* = 0.1766). At 3 h of protocolized ventilation, the PaO₂/FiO₂ ratio was 445.0 [396.0–493.0] and 359.4 [277.9–426.4] in VCV and PCV animals, respectively (*p* = 0.0745). Compliance at 3 h was 18.6 mL/cmH₂O [16.0–21.9] in VCV animals and 18.0 [16.5–22.2] in PCV animals (*p* = 0.9626). Spontaneous effort rates, averaged over 0, 1, 2, and 3 h, were significantly higher in VCV animals (44.0 breaths/min [38.8–51.9]) compared to PCV animals (30.5 [27.4–41.0], *p* = 0.0267). Minute ventilation, also averaged across 0, 1, 2, and 3 h, was higher in VCV vs. PCV animals (20.7 L/min [15.7–24.5] vs. 9.9 [8.3–10.9], *p* = 0.0016). The breath stacking ratio was comparable between groups (VCV: 0.50 [0.41–0.51] vs. PCV: 0.57 [0.52–0.58], *p* = 0.200), confirming adherence to the target breath stacking range (40–70%). To achieve the target breath stacking ratio, a higher flow of inhaled CO₂ was required in VCV vs. PCV animals (3.0 L/min [2.8–3.5] vs. 2.1 [2.0–2.3], *p* = 0.0153). The 3-h average arterial carbon dioxide tension (PaCO₂) was 63.2 [59.8–81.0] mmHg in VCV animals and 55.0 [54.1–61.9] mmHg in PCV animals (*p* = 0.0927).

### Representative waveforms of double triggering in VCV and PCV

**Fig. 1:**
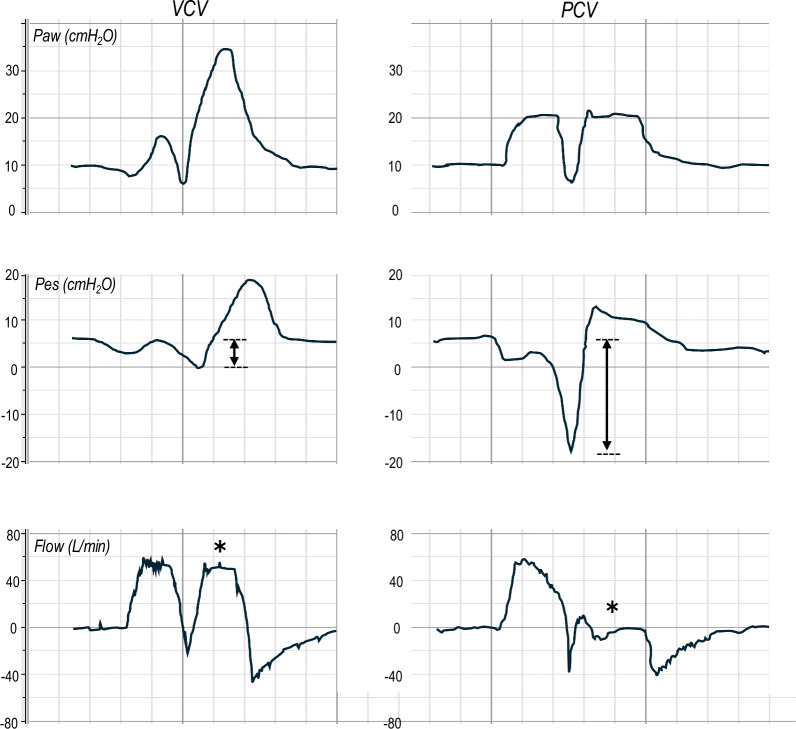
Representative airway pressure, esophageal pressure, and flow waveforms during breath stacking in VCV (*left*) and PCV (*right*). “Airway pressure (Paw), esophageal pressure (Pes), and flow are shown for each mode. *Asterisks* on the flow waveform indicate the additional flow delivered after the second trigger (breath stacking). In VCV (*left*), as evidenced by the flow waveform, breath stacking results in delivery of an additional full tidal volume. In contrast, in PCV (*right*), although the preset airway pressure (PEEP + inspiratory pressure) is maintained, the volume delivered after the second trigger depends on the patient’s inspiratory effort; if the effort has already ceased after the second trigger, there is minimal or no additional flow—and thus no further volume delivery. *Double-headed arrows* on the Pes waveform indicate ΔPes, which is greater in PCV than in VCV, illustrating stronger inspiratory effort in PCV animals.” *VCV* volume-controlled ventilation, *PCV* pressure-controlled ventilation

Figure 1 illustrates representative waveforms of breath stacking during VCV and PCV, highlighting their distinct mechanical consequences. In VCV, each stacked breath delivers a full tidal volume, whereas in PCV, the second trigger prolongs the inspiratory time with minimal additional tidal volume. As indicated by the larger ΔPes in PCV, inspiratory effort was greater than that in VCV in this experiment, as shown later in the quantitative results.

### Risk factors of baro-volutrauma

**Fig. 2:**
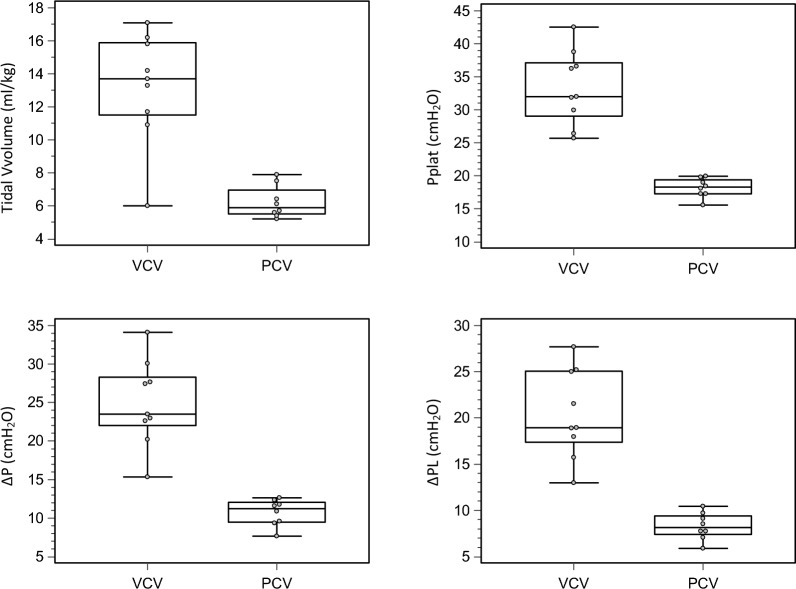
Comparison of respiratory mechanics between VCV and PCV groups during breath stacking. Box plots comparing key respiratory mechanics parameters between VCV and PCV groups during breath stacking. Parameters include tidal volume (*top left*), airway plateau pressure (Pplat, *top right*), airway driving pressure (ΔPaw, *bottom left*), and transpulmonary driving pressure (ΔPL, *bottom right*). Each *dot* represents a single animal. Median and interquartile ranges are displayed. All values were significantly higher in the VCV group, indicating greater mechanical stress during breath stacking compared to PCV. *VCV* volume-controlled ventilation, *PCV* pressure-controlled ventilation, *Paw_plt* airway plateau pressure, *ΔPaw* airway driving pressure (Paw_plt − PEEP), *ΔPL* transpulmonary driving pressure

Assessment of respiratory mechanics during breath stacking is shown in Table 1. All measured parameters were significantly higher in VCV animals compared to PCV animals, including tidal volume (13.3 [11.7–15.8] vs. 6.0 [5.5–7.5] mL/kg, *p* < 0.001), airway plateau pressure (Pplat: 32.0 [29.9–36.6] vs. 18.3 [17.3–19.2] cmH₂O, *p* < 0.001), driving pressure (ΔPaw: 23.5 [22.6–27.7] vs. 11.2 [9.5–11.9] cmH₂O, *p* < 0.001), transpulmonary plateau pressure (PLplat: 20.1 [17.7–28.1] vs. 8.8 [7.4–11.3] cmH₂O, *p* < 0.001), and transpulmonary driving pressure (ΔPL: 19.0 [18.0–25.0] vs. 8.1 [7.6–9.2] cmH₂O, *p* < 0.001) (Table 1 and Fig. 2). In the VCV group, the proportion of animals exceeding thresholds for baro-volutrauma was as follows: tidal volume > 10 mL/kg in 88.9%, Pplat > 30 cmH₂O in 66.7%, ΔPaw > 15 cmH₂O in 100.0%, PLplat > 20 cmH₂O in 55.6%, and ΔPL > 10 cmH₂O in 100.0%. In contrast, these thresholds were exceeded in 0.0 to 12.5% of PCV animals (all *p* < 0.02). The total number of baro-volutrauma risk factors per animal was significantly higher in VCV (4.0 [3.8–5.0]) than in PCV (0.0 [0.0–0.0], *p* < 0.001; Table 1).

**Table 1:** Respiratory mechanics and baro-volutrauma risk factors during breath stacking (averaged over 0–3 h)

Baro-volotrauma risk factors	VCV group (*n* = 9)	PCV group (*n* = 8)	*p*
Pplat (cmH_2_O)	32.0 [29.9–36.6]	18.3 [17.3–19.2]	<0.001
Pplat > 30 (*n* [%])	6.0 [66.7]	0.0 [0.0]	0.0053
ΔP (cmH_2_O)	23.5 [22.6–27.7]	11.2 [9.5–11.9]	<0.001
ΔP > 15 (*n* [%])	9.0 [100]	0.0 [0.0]	<0.001
PLplat (cmH_2_O)	20.1 [17.7–28.1]	8.8 [7.4–11.3]	<0.001
PLplat > 20 (*n* [%])	5.0 [55.6]	0.0 [0.0]	0.0149
ΔPL (cmH_2_O)	19.0 [18.0–25.0]	8.1 [7.6–9.2]	<0.001
ΔPL > 10 (*n* [%])	9.0 [100]	1.0 [12.5]	<0.001
Tidal volume (mL/Kg)	13.3 [11.7–15.8]	6.0 [5.5–7.5]	<0.001
Tidal volume > 10 (*n* [%])	8.0 [88.9]	0.0 [0.0]	<0.001
Total risk factors > threshold	4.0 [3.8–5.0]	0.0 [0.0–0.0]	<0.001

*Pplat* airway plateau pressure, *ΔP* airway driving pressure, *PLplat* plateau transpulmonary pressure, *ΔPL* transpulmonary driving pressure, *BW* body weight

Data represent median [interquartile range] or *n*. Measurements were obtained during spontaneous breathing with breath stacking and averaged across 0, 1, 2, and 3 h of protocolized ventilation

Thresholds used to define barotrauma/volutrauma risk were: Pplat > 30 cmH₂O, ΔP > 15 cmH₂O, PLplat > 20 cmH₂O, ΔPL > 10 cmH₂O, and tidal volume > 10 mL/kg. ‘Total risk factors > threshold’ indicates the number of variables exceeding the predefined thresholds per animal

### Risk factors of atelectrauma

For atelectrauma (Table 2), PCV animals had higher rates of adverse physiological markers: PLexp < −0.5 cmH₂O in 50.0%, SI < 0.9 in 50.0%, ΔPes > 5 cmH₂O in 100.0%, and Pmus > 8 cmH₂O in 87.5%. In comparison, VCV animals showed lower proportions: 11.1, 22.2, 44.4, and 44.4%, respectively (with *p*-values ranging from 0.015 to 0.245). The number of atelectrauma risk factors per animal was significantly higher in the PCV group (3.0 [2.5–3.0]) than in the VCV group (1.0 [0.0–2.0], *p* = 0.0044; Table 2).

**Table 2:** Physiological markers and atelectrauma risk factors during breath stacking (averaged over 0–3 h except for stress index)

Atelectrauma risk factors	VCV group (*n* = 9)	PCV group (*n* = 8)	*p*
PLexp (cmH_2_O)	0.8 [0.2–2.7]	0.8 [−1.2–5.0]	0.7001
PLexp negative (*n* [%])	1.0 [11.1]	4.0 [50.0]	0.0883
ΔPes (cmH_2_O)	4.1 [3.2–6.6]	7.0 [6.2–8.7]	0.0464
ΔPes > 5 (*n* [%])	4.0 [44.4]	8.0 [100.0]	0.0149
Pmusc (cmH_2_O)	7.8 [4.9–9.7]	10.6 [8.6–12.2]	0.0592
Pmusc > 8 (*n* [%])	4.0 [44.4]	7.0 [87.5]	0.0718
Stress index	1.0 [0.9–1.0]	0.9 [0.8–1.0]	0.1472
Stress index < 0.9 (*n* [%])	2.0 [22.2]	4.0 [50.0]	0.2454
Total risk factors > threshold	1.0 [0.0–2.0]	3.0 [2.5–3.0]	0.0044

*SI* stress index, *PLexp* end-expiratory transpulmonary pressure, *ΔPes* esophageal pressure swing, *Pmus* inspiratory muscle pressure

Data represent median [interquartile range] or *n*. All values were averaged over 0, 1, 2, and 3 h during spontaneous breathing with breath stacking

Risk thresholds were defined as follows: PLexp < −0.5 cmH₂O, ΔPes > 5 cmH₂O, Pmus > 8 cmH₂O, and stress index (SI) < 0.9. ‘Total risk factors > threshold’ indicates the number of variables exceeding the predefined thresholds per animal. SI was measured only once at the end of protocolized ventilation, whereas all other parameters were averaged across 0, 1, 2, and 3 h

ΔPes was measured during breath stacking; Pmus was estimated from ΔPes and chest wall compliance

### Lung injury

Respiratory mechanics and gas exchange outcomes at the end of the 3-h ventilation period are summarized in Table 3. There were no significant differences in PaO₂/FiO₂ ratio or final compliance values between groups, but the change in compliance from baseline differed significantly: VCV animals showed a positive change (4.0 [−0.7 to 4.5]), while PCV animals exhibited a decrease (−2.3 [−4.3 to −0.1], *p* = 0.0111; Table 3).

**Table 3:** Gas exchange, respiratory compliance, and lung injury scores at the end of protocolized ventilation

Lung injury assessment	VCV group (*n* = 9)	PCV group (*n* = 8)	*p*
PaO_2_/FiO_2_ (mmHg)	413.3 [350.8–475.0]	354.8 [289.3–434.2]	0.3213
Compliance (mL/cmH_2_O)	20.3 [17.9–22.5]	15.4 [14.4–17.9]	0.1996
PaO_2_/FiO_2_ change (mmHg)	−10.0 [−122.6 to 54.3]	6.1 [1.8–22.0]	0.6058
Compliance change (mL/cmH_2_O)	4.0 [−0.7 to 4.5]	−2.3 [−4.3 to −0.1]	0.0111
Lung injury score	2.0 [2.0–4.0]	3.0 [3.0–5.3]	0.169

Data represent median [interquartile range] or *n*. All parameters were measured at the end of the 3-h protocol

Lung injury scores were obtained from blinded histological evaluation of the right lower lobe

Change values represent difference between baseline (0 h) and final (3 h) measurements

**Fig. 3:**
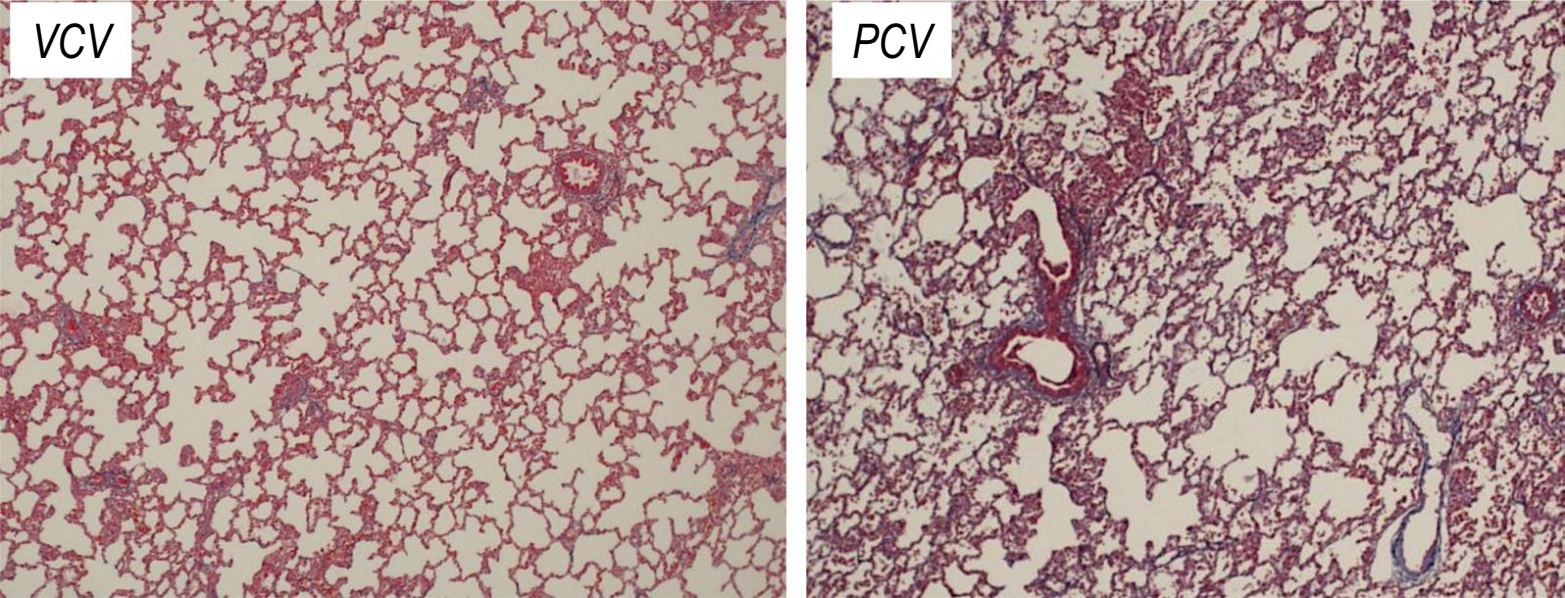
Representative histological lung sections from the VCV and PCV groups. Hematoxylin and eosin-stained sections of the posterior lobe of the right lung, viewed at ×100 magnification. The *left panel* shows a representative sample from the VCV group (lung injury score: 2), and the *right panel* from the PCV group (lung injury score: 3). Both sections exhibit mild alveolar congestion. In the PCV group, focal neutrophil infiltrates are also observed, suggesting greater histological lung injury compared to the VCV group. *VCV* volume-controlled ventilation, *PCV* pressure-controlled ventilation

Histological lung injury scores were comparable between groups, though numerically higher in PCV animals (median 3.0 [3.0–5.3]) than in VCV (2.0 [2.0–4.0], *p* = 0.169; Cliff’s delta = −0.403) (see Table 3, Supplementary Table 1, and Fig. 3).

## Discussion

Our main findings were as follows: (1) under the condition of continuous CO₂ inhalation with strong inspiratory efforts and frequent breath stacking, animals exhibited distinct patterns of spontaneous effort depending on the ventilator mode: VCV was associated with a higher respiratory rate and weaker efforts, whereas PCV was characterized by a lower rate but stronger inspiratory efforts; (2) VCV animals demonstrated more indicators of overdistension-related injury (e.g., large tidal volumes and high plateau pressures), whereas PCV animals showed greater signs of intra-tidal recruitment and atelectrauma; (3) lung injury was comparable—or possibly more severe—in PCV animals, which exhibited more markers of atelectrauma risk, than in VCV animals, which showed increased risk factors for baro-volutrauma.

To our knowledge, this is the first study to experimentally model and quantify lung injury caused by strong inspiratory effort-induced breath stacking—a clinically relevant phenomenon that, until now, lacked a standardized preclinical model. By dynamically adjusting the flow of inhaled CO₂ and ventilator settings, our protocol successfully replicated the conditions under which frequent spontaneous breath stacking occurs in ICU patients.

The distinct injury mechanisms paralleled the different respiratory patterns promoted by hypercapnia-induced high respiratory drive: in VCV, spontaneous efforts occurred at a very high rate with moderate amplitude, whereas in PCV, the rate was lower but the amplitude markedly greater. These differences can be attributed to the varying impact of double triggering on CO₂ elimination. In VCV, double triggering generated true “double-volume” breath stacking, which nearly doubled minute ventilation and therefore required a higher inhaled CO₂ flow to maintain hypercapnia and keep the breath stacking ratio within the target range. To enhance CO₂ clearance, VCV animals primarily increased their respiratory rate rather than inspiratory effort, likely because even moderate efforts generated large tidal volumes. In contrast, in PCV, double triggering did not by itself produce high-volume breaths. As a result, PCV animals compensated for CO₂ inhalation mainly by increasing the strength of inspiratory efforts to augment tidal volume. Baseline measurements obtained 10 min after initiating protocolized ventilation indicated that substantial differences in inspiratory effort had already emerged, suggesting that the ventilator mode rapidly influenced the pattern of respiratory drive. This was further supported by ΔPes dynamics: in the PCV group, ΔPes remained significantly elevated throughout the experimental period, reflecting persistently strong inspiratory efforts, whereas in the VCV group, ΔPes gradually declined over time (Supplementary Fig. 2). These findings suggest that the choice of ventilatory mode may influence the development and modulation of respiratory drive, even under otherwise standardized experimental conditions.

Consistent with our hypothesis, mechanical stress and strain parameters—including tidal volume, airway and transpulmonary plateau pressures, and driving pressures—were significantly elevated in the VCV group during breath stacking, frequently exceeding accepted lung-protective thresholds. This reaffirms that double triggering during VCV can impose a high mechanical burden. In contrast, PCV animals showed no evidence of overdistension (plateau pressures, driving pressures, and tidal volumes remained within protective ranges), yet injury severity was comparable to that observed in VCV, with a trend toward greater damage. Physiological indicators—including persistently high ΔPes, low stress index, and negative PLexp—suggest that vigorous inspiratory efforts, combined with insufficient PEEP, promoted intra-tidal recruitment and atelectrauma. However, because we did not directly evaluate collapse and reopening of the lung regions with imaging tools such as CT or EIT, these interpretations should be considered hypothesis-generating for future mechanistic studies.

Although derived from a porcine model, these findings may have important clinical implications for patients with strong respiratory drive. First, our data suggest that the choice of ventilatory mode can influence how patients respond to hypercapnia: under VCV, minute ventilation tends to increase through a rise in respiratory rate, whereas under PCV, patients are more likely to enhance their inspiratory effort to achieve similar CO₂ clearance. This differential response may have direct consequences for lung injury. In VCV, the combination of strong drive and frequent double triggering leads to large tidal volumes and high airway pressures, raising the risk of overdistension and barotrauma. In contrast, PCV may appear more lung-protective by avoiding double-volume breath stacking; however, it may promote stronger inspiratory efforts that, especially in the presence of insufficient PEEP, can exacerbate mechanical stress and promote intra-tidal recruitment, increasing the risk of atelectrauma.

Thus, neither mode can be considered inherently safe in the setting of strong spontaneous drive. Rather than debating which ventilation mode is preferable, it is more important to challenge the false sense of safety associated with “controlled” modes. In VCV, breath stacking may nearly double the delivered tidal volume, while in PCV, the actual plateau pressure—reflecting both ventilator pressure and patient effort—can exceed the preset inspiratory pressure. In this context, peak pressure displayed on the ventilator is a poor surrogate for end-inspiratory alveolar pressure, underscoring the need for end-inspiratory hold maneuvers to estimate plateau and driving pressures. Importantly, even when end-inspiratory hold maneuvers indicate airway and transpulmonary pressures within protective ranges, vigorous inspiratory efforts can still promote lung injury. Failure to address such strong inspiratory efforts carries the risk of ongoing injury that may remain clinically unrecognized, despite apparently protective ventilator settings. This highlights that conventional global indices of lung stress and strain may not fully capture the injurious consequences of patient effort, providing the rationale for more refined monitoring approaches.

Our study confirms this limitation, showing that substantial lung injury can be induced by strong inspiratory efforts, even without exceeding the thresholds of global lung stress and strain commonly used in clinical practice. Therefore, it is important to detect—or at least suspect—intra-tidal recruitment and atelectrauma at the bedside. Stress index and tidal lung hysteresis can help identify the presence or absence of intra-tidal recruitment under passive conditions [12, 13]. Accordingly, these metrics may guide ventilator setting adjustments before allowing spontaneous breathing activity. Under active conditions, intra-tidal recruitment is often associated with occult pendelluft, a ventilation pattern that can be detected using electrical impedance tomography (EIT) [6]. Finally, because the primary driver of P-SILI is the patient’s strong inspiratory effort, monitoring spontaneous activity is essential. Strong efforts should be suspected whenever high-drive patient–ventilator asynchronies—such as early cycling and double triggering—are observed on ventilator waveforms [14]. This highlights the need for automated, continuous monitoring of patient–ventilator interaction. When an excessively strong effort is suspected, the pressure generated by the inspiratory muscles should be assessed. Pes measurement is the reference method [15], but reliable estimates of Pmus can be obtained from drops in airway pressure during expiratory hold maneuvers [16].

This study has several limitations. First, the sample size was modest, limiting statistical power, particularly for histological comparisons. Second, because breath stacking frequency was intentionally maintained within a target range (40–70%) by adjusting ventilator and CO₂ settings, we were unable to investigate potential correlations between disease severity (e.g., compliance) and the occurrence or magnitude of breath stacking. Moreover, this strategy resulted in two distinct physiological states: a higher respiratory rate with relatively lower inspiratory effort in the VCV group, and a lower rate with stronger effort in the PCV group. As such, it is difficult to disentangle whether the observed injury differences were mode-specific or primarily reflected differences in respiratory drive. Future studies should consider matching groups by inspiratory effort (e.g., ΔPes) to better isolate the effect of ventilation mode. Third, we did not directly assess tidal recruitment (e.g., using low-flow PV curves, CT, or EIT), and histological sampling was restricted to a single lobe, potentially missing regional heterogeneity. Finally, interspecies differences in drive regulation and respiratory mechanics may limit extrapolation to human ARDS.

In conclusion, our study suggests that breath stacking due to strong inspiratory effort is a form of high-drive asynchrony that can promote lung injury through multiple mechanisms. In VCV, it may deliver excessive tidal volumes and increase the risk of baro-volutrauma, whereas in PCV, it reflects vigorous inspiratory efforts that predispose to intra-tidal recruitment and atelectrauma, particularly in the presence of pre-existing lung injury or insufficient PEEP. Therefore, the detection of breath stacking should prompt clinicians to evaluate and mitigate risk factors for ventilator-induced lung injury. Further clinical studies are warranted to confirm these experimental findings and refine ventilatory strategies for ARDS patients with strong respiratory drive.

## Supplementary Information

Additional file 1: Supplementary Figure 1. Protocol for adjusting ventilator settings to achieve a breath stacking ratio of 40–70%. Stepwise protocol used to dynamically adjust ventilator settings and inhaled CO₂ to induce strong inspiratory effort and achieve the target breath stacking ratio in both VCV and PCV modes. The protocol includes adjustments to inspiratory flow, inspiratory time, PEEP, and tidal volume, tailored to each ventilation mode. If the target ratio was not reached, the cycle was repeated from Step 1. VCV: volume-controlled ventilation; PCV: pressure-controlled ventilation.

Additional file 2: Supplementary Figure 2. Time course of ΔPes in VCV and PCV groups. Line plots showing the time course of four key respiratory mechanics parameters—tidal volume, airway plateau pressure (Paw_plt), transpulmonary driving pressure (ΔPL), and airway driving pressure (ΔPaw)—measured during breaths with breath stacking at baseline (0 hour) and after 3 hours of mechanical ventilation. Data are stratified by ventilator mode: volume-controlled ventilation (VCV, blue) and pressure-controlled ventilation (PCV, red). Shaded areas represent the 95% confidence intervals. Across all parameters, VCV was associated with persistently higher values throughout the study period, indicating greater mechanical stress and strain compared to PCV.

Additional file 3.

## Data Availability

The datasets generated and/or analyzed during the current study are available from the corresponding author upon reasonable request.
