# Supplementary material for: Lung injury promoted by strong inspiratory efforts and breath stacking: impact of ventilation mode
Source: Intensive Care Med Exp. 2025 Oct 29;13:110. doi: 10.1186/s40635-025-00821-0 (PMC12572466; doi:10.1186/s40635-025-00821-0)
Supplement: Supplementary file 1 — Additional file 1: Supplementary Figure 1. Protocol for adjusting ventilator settings to achieve a breath stacking ratio of 40–70%. Stepwise protocol used to dynamically adjust ventilator settings and inhaled CO₂ to induce strong inspiratory effort and achieve the target breath stacking ratio in both VCV and PCV modes. The protocol includes adjustments to inspiratory flow, inspiratory time, PEEP, and tidal volume, tailored to each ventilation mode. If the target ratio was not reached, the cycle was repeated from Step 1. VCV: volume-controlled ventilation; PCV: pressure-controlled ventilation. [file 40635_2025_821_MOESM1_ESM.docx]

# Supplementary Figure 1

# Protocol for Adjusting Ventilator Settings to Achieve a Breath Stacking Ratio of 40–70%.

1. Start inhaled CO₂ at 2 L/min and adjust as needed to achieve sufficient inspiratory effort for breath stacking.

2. Adjust the I:E ratio:
 - In VCV: adjust the inspiratory flow rate.
 - In PCV: adjust the inspiratory time.

3. Adjust PEEP:
 - Higher PEEP levels tend to decrease inspiratory effort but prolong inspiratory time in pigs.

4. Adjust trigger sensitivity

5. If target not achieved → Return to Step 1.
