# Supplementary figures and images for: Lung injury promoted by strong inspiratory efforts and breath stacking: impact of ventilation mode
Source: Intensive Care Med Exp. 2025 Oct 29;13:110. doi: 10.1186/s40635-025-00821-0 (PMC12572466; doi:10.1186/s40635-025-00821-0)

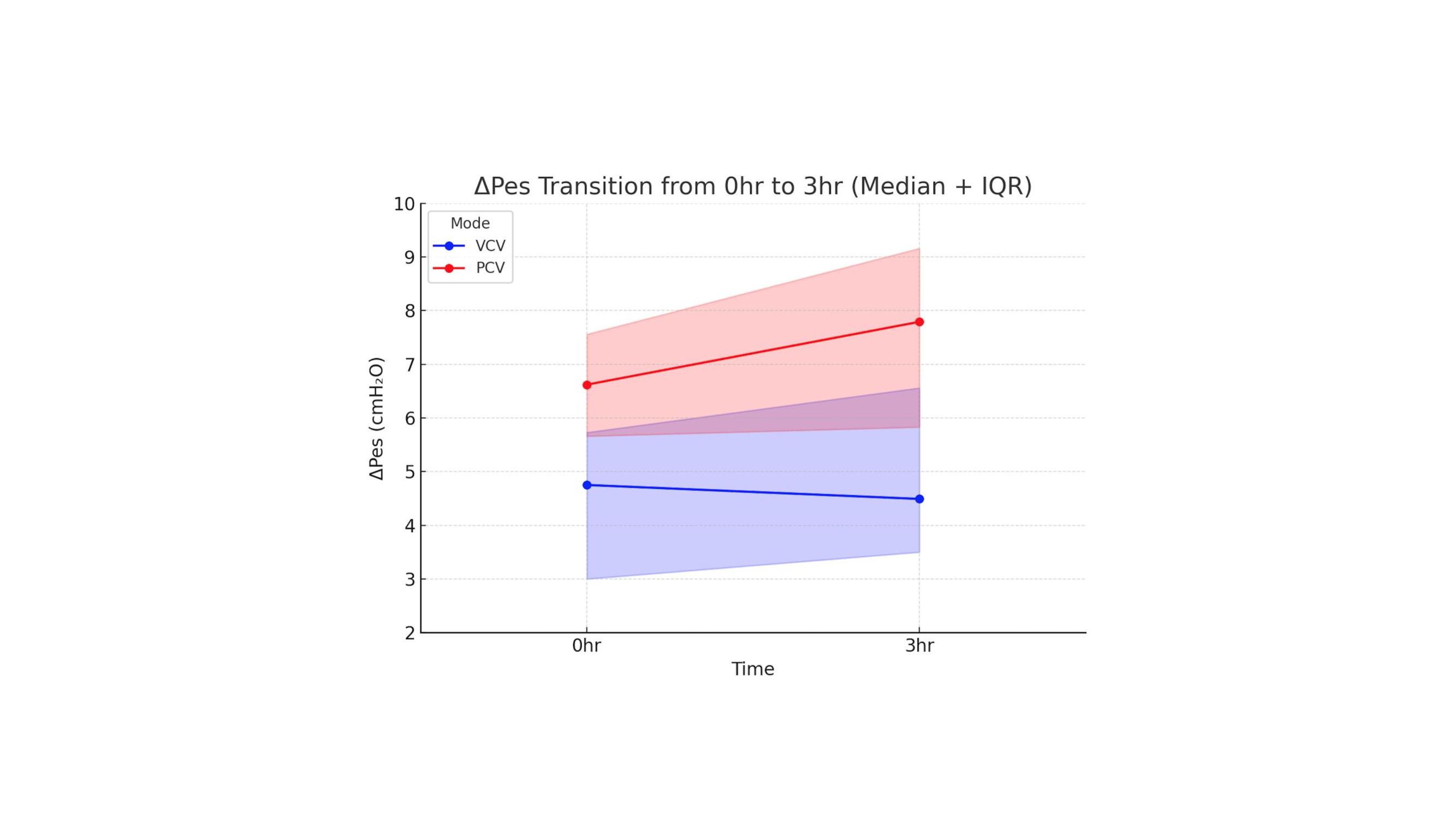

Supplement: Supplementary file 2 — Additional file 2: Supplementary Figure 2. Time course of ΔPes in VCV and PCV groups. Line plots showing the time course of four key respiratory mechanics parameters—tidal volume, airway plateau pressure (Paw_plt), transpulmonary driving pressure (ΔPL), and airway driving pressure (ΔPaw)—measured during breaths with breath stacking at baseline (0 hour) and after 3 hours of mechanical ventilation. Data are stratified by ventilator mode: volume-controlled ventilation (VCV, blue) and pressure-controlled ventilation (PCV, red). Shaded areas represent the 95% confidence intervals. Across all parameters, VCV was associated with persistently higher values throughout the study period, indicating greater mechanical stress and strain compared to PCV. [file 40635_2025_821_MOESM2_ESM.tiff]
