# Supplementary material for: Lung injury promoted by strong inspiratory efforts and breath stacking: impact of ventilation mode
Source: Intensive Care Med Exp. 2025 Oct 29;13:110. doi: 10.1186/s40635-025-00821-0 (PMC12572466; doi:10.1186/s40635-025-00821-0)
Supplement: Supplementary file 3 — Additional file 3. [file 40635_2025_821_MOESM3_ESM.docx]

Supplementary Table 1. Individual histological lung injury scores and component parameters for each animal.

| **ID** | **mode** | **alveolar congestion** | **Hemorrhage** | **infiltration or aggregation of neutrophils in airspaces or vessel walls** | **Thickness of alveolar wall/hyaline mambrane formation** | **total score** |
| --- | --- | --- | --- | --- | --- | --- |
| 1 | VCV | 0 | 1 | 2 | 0 | 3 |
| 2 | VCV | 0 | 0 | 1 | 0 | 1 |
| 3 | VCV | 1 | 0 | 1 | 0 | 2 |
| 4 | VCV | 1 | 0 | 1 | 0 | 2 |
| 5 | VCV | 1 | 0 | 1 | 0 | 2 |
| 6 | VCV | 2 | 1 | 4 | 0 | 7 |
| 7 | VCV | 1 | 1 | 2 | 0 | 4 |
| 8 | VCV | 1 | 1 | 2 | 0 | 4 |
| 9 | VCV | 0 | 0 | 1 | 0 | 1 |
| 10 | PCV | 4 | 1 | 4 | 0 | 9 |
| 11 | PCV | 2 | 1 | 2 | 0 | 5 |
| 12 | PCV | 1 | 0 | 2 | 0 | 3 |
| 13 | PCV | 1 | 0 | 2 | 0 | 3 |
| 14 | VCV | 3 | 0 | 3 | 0 | 6 |
| 15 | PCV | 1 | 0 | 2 | 0 | 3 |
| 16 | PCV | 1 | 0 | 1 | 0 | 2 |
| 17 | PCV | 1 | 1 | 1 | 0 | 3 |
